# Refinement of Molecular Diagnostic Protocol of Auditory Neuropathy Spectrum Disorder: Disclosure of Significant Level of Etiologic Homogeneity in Koreans and Its Clinical Implications

**DOI:** 10.1097/MD.0000000000001996

**Published:** 2015-10-30

**Authors:** Mun Young Chang, Ah Reum Kim, Nayoung K.D. Kim, Chung Lee, Woong-Yang Park, Byung Yoon Choi

**Affiliations:** From the Department of Otorhinolaryngology, Seoul National University Hospital, Seoul National University College of Medicine (MYC, ARK); Samsung Genome Institute, Samsung Medical Center, Seoul (NKDK, CL, WYP); Department of Health Sciences and Technology, SAIHST, Sungkyunkwan University (CL); Department of Molecular Cell Biology, Sungkyunkwan University School of Medicine, Suwon (WYP); and Department of Otorhinolaryngology, Seoul National University Bundang Hospital, Seongnam, Republic of Korea (BYC).

## Abstract

Auditory neuropathy spectrum disorder (ANSD) is a sensorineural hearing disorder caused by dysfunction of auditory neural conduction. ANSD has a heterogeneous etiology, including genetic factors; the response to cochlear implantation significantly varies depending on the etiology. The results of timely cochlear implantation for *OTOF*-related ANSD (DFNB9) have been reported to be good. Therefore, identifying the causative gene of ANSD, especially *OTOF*, is an important issue to rehabilitate these patients.

Six sporadic ANSD subjects without anatomical abnormality of the cochlear nerve, including the 4 subjects that were previously reported to be without detectable *OTOF* mutation, were included. We performed targeted resequencing (TRS) of known deafness genes and multiphasic bioinformatics analyses of the data that ensured detection of capture failure and structural variations. Exclusion of SNP was also double checked. The TRS data previously obtained from 2 subjects were reanalyzed. Through this study, we detected 2 mutant alleles of *OTOF* from 5 (83.3%) of 6 ANSD subjects. All of the 5 subjects carried at least 1 mutant allele carrying p.R1939Q. This variant was categorized as a simple SNP (rs201326023) in the database and it resided in the exon with frequent capture failures, which previously led to exclusion of this variant from eligible candidacy mistakenly. In addition, we detected a structural variation within *OTOF* from a previously undiagnosed ANSD subject, which was the second structural variation reported in DFNB9 subjects to date.

We identify a strong etiologic homogeneity of prelingual ANSD in case of the anatomically normal cochlear nerve in Koreans and now report DFNB9 as the single overwhelming cause. Multiphasic analysis of TRS data ensuring detection of capture failure and structural variations would be expected to reveal DFNB9 from a substantial portion of previously undiagnosed ANSD subjects in Koreans. Based on our results, we propose a novel strategy that incorporates imaging studies, prevalent mutation screening and multiphasic analysis of TRS data in a stepwise manner to correctly detect DFNB9 in Koreans.

## Abstract 2

Supplemental Digital Content is available in the text

## INTRODUCTION

Auditory neuropathy spectrum disorder (ANSD) is a frequently detected sensorineural hearing disorder characterized by the presence of otoacoustic emissions (OAE) and severe abnormality of auditory pathways in audiologic tests, revealing dysfunctional neural conduction of auditory pathway despite intact outer hair cell function.^1,2^ About 10% of infants that are diagnosed as having profound hearing loss suffer from ANSD.^3,4^ Several factors, including perinatal hypoxia, infection, and genetic factors could cause ANSD.^4–8^ Prelingual nonsyndromic ANSD is closely associated with genetic factors. *GJB2*, *PJVK*, *OTOF*, and *DIAPH3* have been identified as causative genes.^9–11^ In general, patients with ANSD are thought to respond poorly to cochlear implant due to weak stimulability of the auditory nerve.^12,13^ However, the lesion of ANSD could be located in various sites, including the inner hair cells, synapses between the inner hair cells and auditory nerve terminals or auditory nerve.^14^ The stimulability of the auditory nerve varies depending on the location of the lesion. Loundon et al divided ANSD into 2 types: isolated endocochlear hearing loss and real neuropathies. They supposed that isolated endocochlear hearing loss would have normal stimulability of the auditory nerve, and recommended cochlear implantation to isolated endocochlear hearing loss.^15^ Therefore, it is important to distinguish isolated endocochlear hearing loss from other ANSD.

*OTOF-*related ANSD (DFNB9) is regarded as a representative endocochlear hearing loss. *OTOF* encodes otoferlin, which is thought to play a crucial role in the exocytosis of vesicles at the auditory inner hair cell synapses. Several studies suggested otoferlin as the major Ca^2+^ sensor-triggering membrane fusion protein at the inner hair cell synapse,^16–18^ while other studies suggested otoferlin to interact with Rab8b to recycle endosomes and transport vesicles.^19,20^ Therefore, we predict that DFNB9 deafness could be attributed to dysfunction in signal transmission between the inner hair cells and auditory nerve terminals, but intact stimulability of the auditory nerve. Indeed, Rouillon et al^21^ showed good results after cochlear implantation in DFNB9 subjects. In their study, 2 subjects underwent cochlear implantation at the age of 35 months and 4 years, respectively. One subject showed 100% of identification on open-set words and 60% of identification on open-set sentences at 36 months postsurgery. The other subject showed 50% of identification on open-set words and 45% of identification on open-set sentences at 18 months postsurgery. These data supported that DFNB9 is a representative endocochlear hearing loss, and early cochlear implantation should strongly be considered. Conversely, Madden et al^22^ reported that hyperbilirubinemia was associated with spontaneous improvement of ANSD, and a stable audiogram was achieved by the age of 18 months. Sequentially, in such a case, it was suggested that cochlear implantation should be held until the age of 18 months. Therefore, it is very important to distinguish DFNB9 from other ANSD, and *OTOF* mutations could be an important biomarker that guarantees a favorable prognosis after cochlear implantation in ANSD subjects. Given this, early bilateral simultaneous cochlear implantation can be justified in DFNB9 subjects, warranting a timely and cost-effective detection of *OTOF* mutations.

In this study, we propose a hierarchical and multiphasic molecular diagnostic approach to ANSD subjects in Korea based on our experience. In addition, we report a remarkably high prevalence of DFNB9 among Korean ANSD subjects with anatomically normal cochlear nerve, which is, in part, contrary to previous reports from Korea.

## METHODS

### Subjects and Ethical Considerations

All procedures in this study were approved by the institutional review boards at Seoul National University Hospital (IRBY-H-0905-041-281) and Seoul National University Bundang Hospital (IRB-B-1007-105-402). Written informed consent was obtained from all subjects or guardians in case of children. Six families (SH81, SH132, SB10, SB22, SB42, and SB204), whereby ANSD was segregated in a sporadic or an autosomal recessive fashion, were included in this study between June 2010 and March 2015. Among these families, 4 families (SB10, SB42, SB22, and SH132) were previously reported to carry no *OTOF* mutation by TRS-134 (SB10–23 and SB132–273) or Sanger sequencing of *OTOF* (SB22–51 and SB42–79). Three members over 2 generations from each family, at minimum, were identified and evaluated at Seoul National University Hospital and Seoul National University Bundang Hospital for this study. Phenotype evaluations included medical and developmental history interviews, physical examinations, and audiometric evaluation.

### Audiometric Evaluation and Anatomical Evaluation of the Cochlear Nerve

Auditory brain stem response threshold (ABRT), distortion product otoacoustic emission (DPOAE), and transient-evoked otoacoustic emission (TEOAE) tests were carried out on SH81–185, SH132–273, SB10–23, SB22–51, SB42–79, and SB204–398. Internal auditory canal magnetic resonance imaging (MRI) was used to identify any inner ear anomalies related to hearing loss, including anatomical abnormality of the cochlear nerve. When internal auditory canal MRI was not available, temporal bone computed tomography (CT) was used.

### Molecular Genetic Testing

Blood samples were taken from 4 subjects (SH81–185, SB22–51, SB42–79, and SB204–398) and genomic DNAs were extracted from peripheral blood. Targeted resequencing of the known 134 deafness genes (TRS-134) from these subjects was done by Otogenetics (Norcross, GA).^23^ Then, the acquired readings were aligned to UCSC hg19 reference genome and variants were obtained. The TRS data previously obtained from SB10–23 and SH132–273 were analyzed and filtered again. Furthermore, bioinformatics analyses were performed as previously described.^23^ In brief, these data were filtered through 2 steps to select candidate SNPs in nonsyndromic sensorineural hearing loss (NSHL) genes.

**FIGURE 1:**
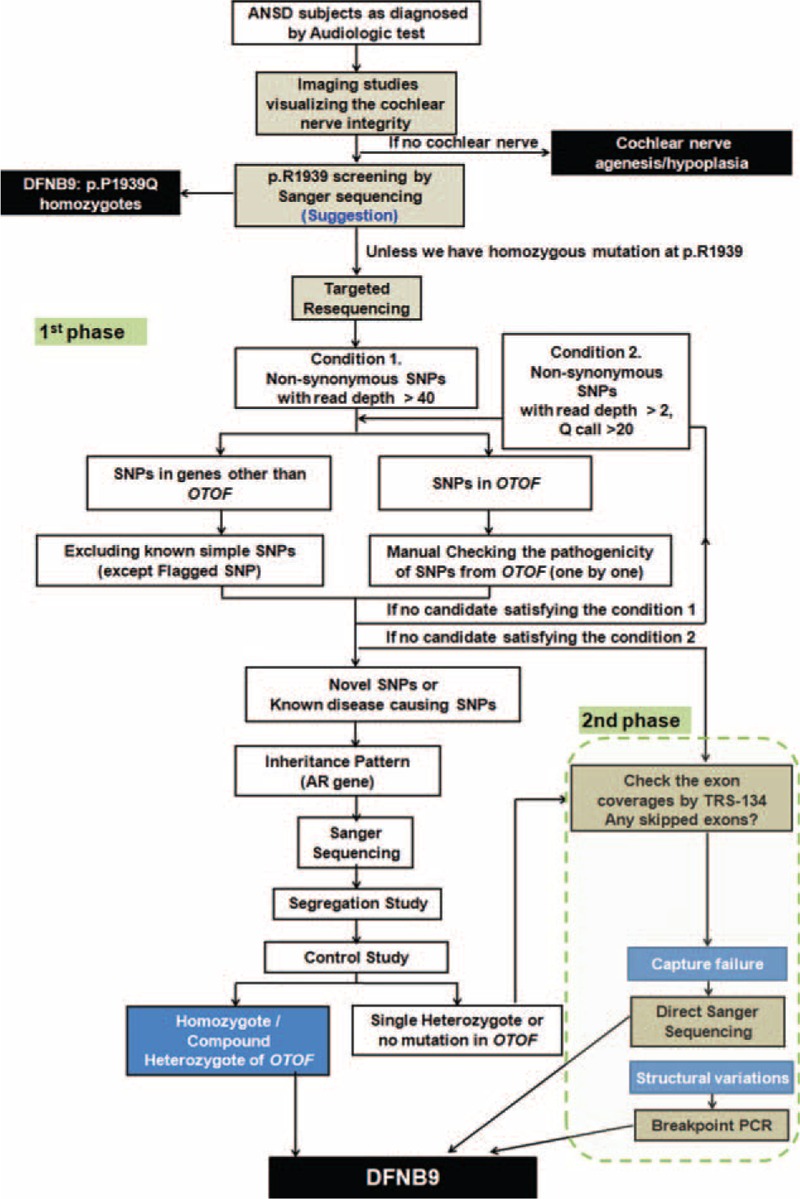
Hierarchical and multiphasic diagnostic pipeline. This iagnostic strategy involves imaging, screening of a prevalent mutation, and multiphasic analysis of TRS data for etiologic diagnosis of auditory neuropathy spectrum disorder. AR = autosomal recessive, PCR = polymerase chain reaction, SNP = single-nucleotide polymorphism, TRS = targeted resequencing.

During the first phase of the filtering process, nonsynonymous SNPs were mainly targeted. Nonsynonymous SNPs with a depth of more than 40 were initially selected. Selected SNPs were compared with the Single Nucleotide Polymorphism database (dbSNP build 138) and with the in-house database, which is an independent cohort comprised of 54 normal Korean subjects. Known simple SNPs, except flagged SNPs, were excluded. Exceptionally, SNPs from the *OTOF* gene were checked one by one before exclusion to prevent omitting candidate variants of *OTOF* gene. In case of having no convincing candidate variants after the first run of the filtering process, we loosened the selection criteria of the coverage depth and Q score to >2 and >20%, respectively. In the following step, we checked the inheritance pattern of affected subjects, and excluded SNPs that were not matched with the affected subject's inheritance pattern. Then, we validated the filtered SNPs in parents of each subject by Sanger sequencing and checked additional 426 unrelated Korean control chromosomes for filtered SNPs (Fig. 1). Pathogenicity of the missense variants was predicted using SIFT and Polyphen-2. For an estimation of the evolutionary conservation of the amino acid sequence, we referred to GERP++ score in UCSC genome browser (http://genome.ucsc.edu/).

For the cases with inconclusive molecular diagnosis after the first phase of TRS-134 analysis, we performed the second phase of analysis, which focused on the detection of structural variations or capture failure, especially in *OTOF*. Firstly, in order to differentiate the regions that were not captured by TRS from the loci of hetero- or homozygote deletions, we checked whether the sequencing readings in *OTOF* were evenly covered. Secondly, for the well-covered regions, we searched for split or discordant readings that supported structural variations or breakpoints, for not only exons, but also introns in *OTOF* by IGV (Integrative Genomic Viewer, http://www.broadinstitute.org/igv/home). If we found a clue to the presence of a large genomic deletion in the TRS-134 data, we performed breakpoint polymerase chain reaction (PCR) to confirm a genomic deletion. In case of suspicion of poor coverage over the certain exon of *OTOF*, we performed a Sanger sequencing of the exon that was not sufficiently covered by TRS-134 (Figure 1).

## RESULTS

### Auditory Phenotype

**FIGURE 2:**
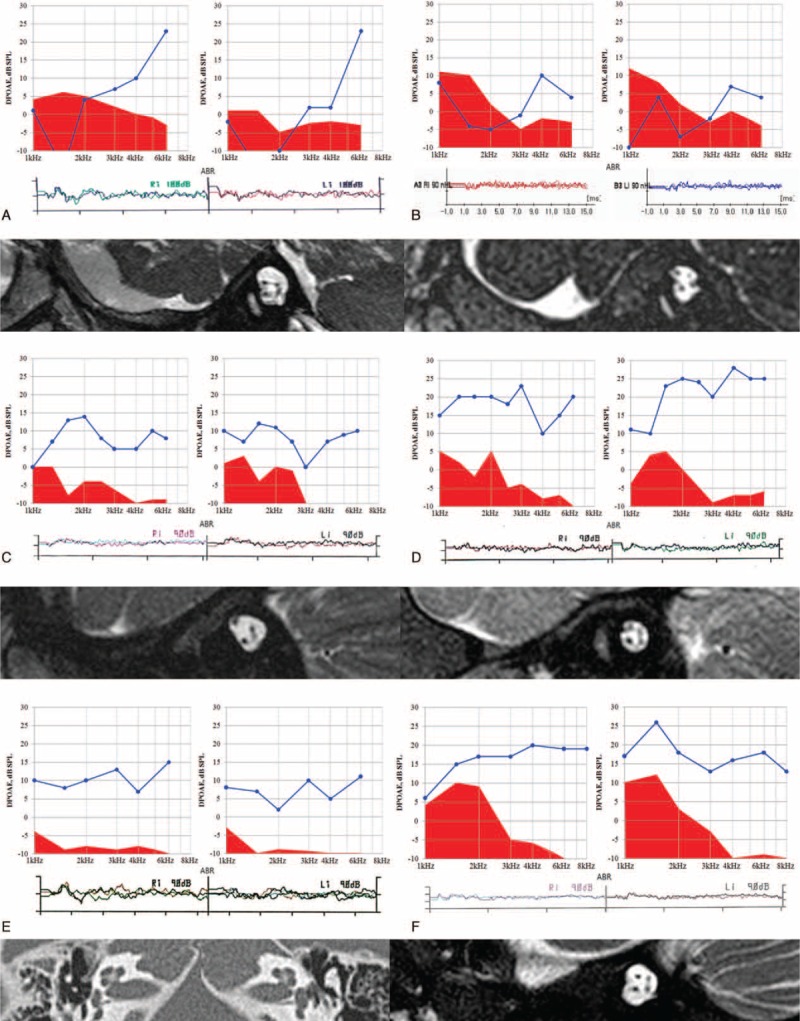
Clinical features suggesting auditory neuropathy spectrum disorder with the intact cochlear nerve. Audiologic results from 6 probands (A: SH81–185, B: SH132–273, C: SB10–23, D: SB22–51, E: SB42–79, F: SB204–398) show normal otoacoustic emission (OAE) response but no response to 90 or 100 dB of click sound in the auditory brainstem response tests. Internal auditory canal magnetic resonance images from 5 probands (A: SH81–185, B: SH132–273, C: SB10–23, D: SB22–51, F: SB204–398) show the intact cochlear nerve. Temporal bone computed tomography of SB42–79 (E) revealed no narrow bony cochlear nerve canal.

Six subjects, SH81–185, SH132–273, SB10–23, SB22–5, SB42–79, and SB204–398, showed no response to 90 or 100 dB click sounds in ABRT testing. The response of DPOAE and TEOAE was present in all 6 subjects (Figure 2), compatible with the clinical diagnosis of ANSD. Parents of all 6 subjects denied any exposure to risk factors, such as drugs or loud noises. No syndromic features were detected in the physical examination. Internal auditory canal MRI clearly revealed no anatomical abnormality of the cochlear nerve in the 5 probands (SH81–185, SH132–273, SB10–23, SB22–5, and SB204–398). In SB42–79, brain MRI was performed instead of internal auditory canal MRI, and it showed a trace of the intact cochlear nerve. Temporal bone CT was performed to visualize the bony cochlear nerve canal in this subject for the purpose of predicting the cochlear nerve status, and revealed a normal-sized bony cochlear nerve canal, which strongly suggests the intact cochlear nerve (Figure 2).

### Targeted Resequencing Data Analysis

Targeted resequencing was newly performed in 4 subjects (SH81–185, SB22–51, SB42–79, and SB204–398), and TRS data, previously obtained from SH132–273 and SB10–23, was revisited. The readings were aligned to a human reference genome. Bioinformatics analyses were carried out as mentioned above (Fig. 1). Then, candidate variants were identified in each proband and validated by Sanger sequencing in their parents, as well as the probands.

**FIGURE 3:**
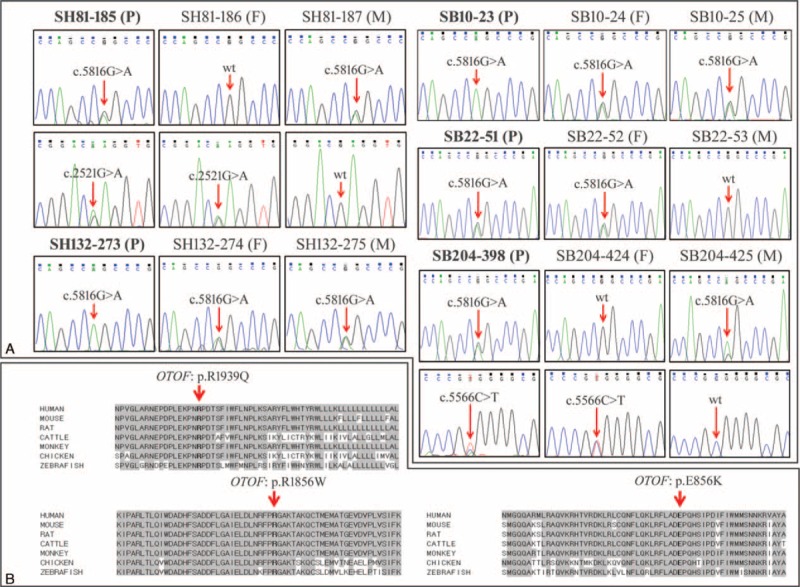
Sanger sequencing traces of variants and conservation of mutant residues (A) Sanger sequencing traces of SH81–185: c.5816G > A (p.R1939Q)/c.2521G > A (p.E856K), SH132–273: c.5816G > A (p.R1939Q) homozygote, SB10–23: c.5816G > A (p.R1939Q) homozygote, SB22–51: c.5816G > A (p.R1939Q)/skipped exon 12, SB204–398: c.5816G > A (p.R1939Q)/c.5566C > T (p.R1856W). (B) Conservation of mutant residues among orthologs from several species. p. R1939, p.E856, and p.R1856 are conserved among human, mouse, rat, cattle, monkey, chicken, and zebrafish. ∗P: proband, F: father, M: mother.

During the first phase of filtration, we were able to make a definitive molecular diagnosis from 2 probands (SH81–185 and SH132–273) and detected at least 1 mutant allele of *OTOF* from 4 of 6 ANSD subjects when we loosened the selection criteria in the coverage and quality score (Table 1). The proportion of regions ×20 coverages among the total of 1737 regions over the 134 deafness genes ranged from 1.15% to 3.17% (mean 2.03 + 0.82%). During this first phase of filtration, we did not exclude known simple SNPs in *OTOF* until their nonpathogenicity was verified (Fig. 1, Table 1, and Supplementary Table S1, http://links.lww.com/MD/A518). Among the simple SNPs in *OTOF*, which would have been excluded without this conservative step, p.R1939Q (rs201326023) merited special attention (Table 1). Three of 4 subjects carried p.R1939Q (p.R1939Q and p.E856K for SH81–185, p. R1939Q homozygote for SH132–273 and p. R1939Q single heterozygote for SB22–51, respectively) and 1 subject carried p.R1856W (p.R1856W single heterozygote for SB204–398) (Table 2 and Figure 3). Even though p.R1939Q variant was previously registered as a simple SNP (rs201326023), this variant, which exclusively affects the cochlear isoform, has been reported to account for ANSD in Japanese and Chinese subjects,^24,25^ strongly suggesting the pathogenicity of this variant. The p.R1939Q, p.E856K, and p.R1856W variants were not found among the 426 control chromosomes from unrelated Koreans with normal hearing. In addition, the SIFT and Polyphen-2 analyses consistently identified *OTOF* p.R1939Q, p.E856K, and p.R1856W as “damaging.” Furthermore, p.R1939, p.E856, and p.R1856 were well-conserved in several species, as indicated by the high GERP++ score of 4.8 and 5.0, respectively (Figure 3). Collectively, this result indicated the pathogenicity of 3 variants.

**TABLE 1:**
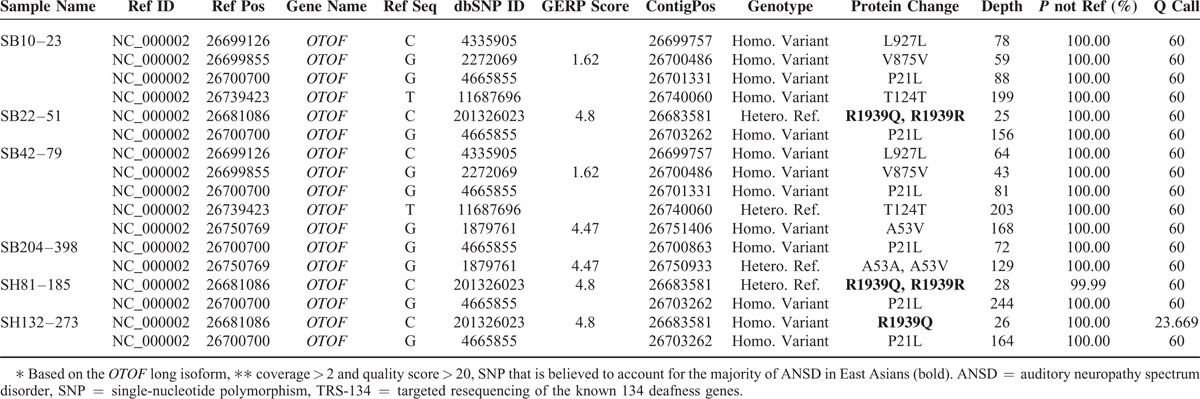
*OTOF* SNP Lists∗ (Excluding Novel or Clinically Associated (Flagged) SNP) Obtained Through TRS-134∗∗ From 6 ANSD Patients: Variants in Coding Regions

**TABLE 2:**
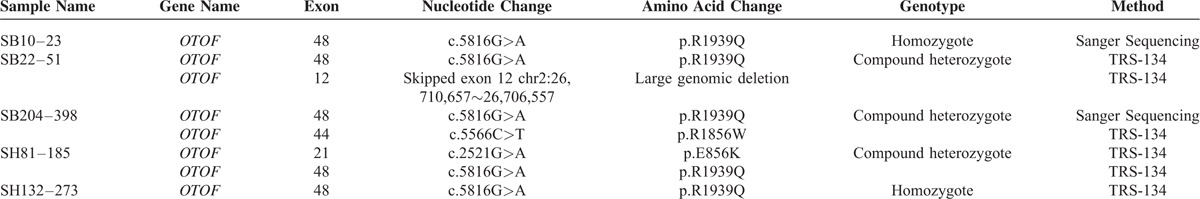
Final Strong Candidates Through Sanger Sequencing or TRS-134 From 5 Auditory Neuropathy Spectrum Disorder Patients

**FIGURE 4:**
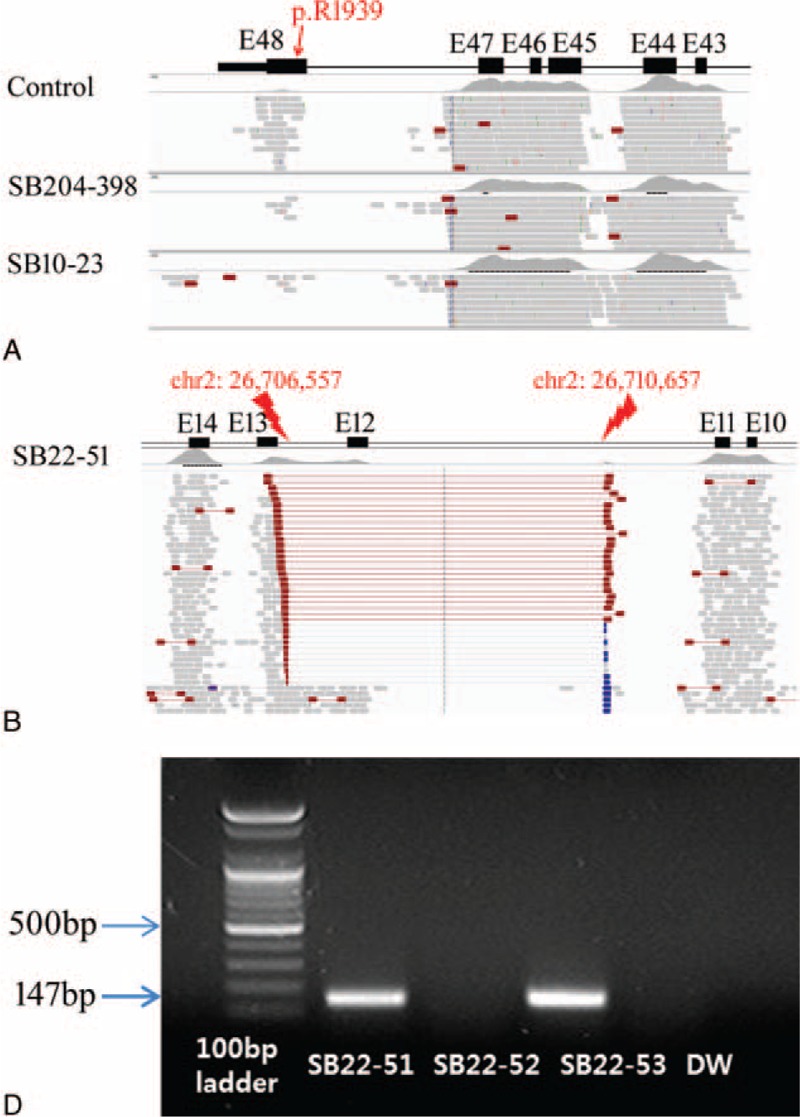
The second phase of TRS-134 data analyses (SB10–23, SB22–51, and SB204–398). (A) Targeted resequencing (TRS) data for SB10–23 and SB204–398 by the IGV viewer. A red box shows TRS capture failure compared with the control. The black box indicates each exon (referred to as “E”). (B) The region where a large genomic deletion is suspected in *OTOF* for SB22–51. Supportive discordant readings are distributed between Exon 11–13. (C) The result of breakpoint polymerase chain reaction (PCR) using our primer set shows genomic deletion of exon 12 in SB22–51 (proband), which is derived from SB22–53 (mother)'s one, while SB22–52 (father) has a normal allele with no deletion as below. The rightmost one is water control. Long range PCR and sequence analysis of PCR product figure out the exact breakpoint (chr2:26,710,657–chr2:26,706,557) with skipped exon 12. TRS-134 = targeted resequencing of the known 134 deafness genes.

Due to the inconclusive status of molecular etiology of the remaining 4 probands (*OTOF* single heterozygote (SB22–51 and SB204–398) and no detectable variant (SB10–23 and SB42–79)) after the initial filtering of TRS-134 results, we inspected the different phases of the TRS-134 data: we checked the exon coverages to detect, if any, capture failures or structural variations, such as large deletions or duplication in and around *OTOF*. Through this second phase of TRS-134 data analyses, we identified that the exon 48, the last exon of *OTOF*, was notably poorly covered except in SH81–185 (Supplementary Table S2, http://links.lww.com/MD/A518). Especially from SB10–23 and SB204–398, we detected a total capture failure involving the exon 48 of *OTOF*, where the predominant variant, p.R1939Q, resides (Figure 4A). Subsequent Sanger sequencing of the exon 48 from SB10–23 and SB204–398 confirmed a homozygous p.R1939Q variant from SB10–23 and a compound heterozygote p.R1939Q and p.R1856W for SB204–398 (Figure 3). From SB22–51 with a single heterozygous p.R1939Q variant as detected by the first phase analysis of TRS-134, we found a region where a large genomic deletion in *OTOF* was strongly suspected (Figure 4B). Subsequent breakpoint PCR confirmed a large genomic deletion (chr2:26,710,657∼26,706,557) from SB22–51 (Figure 4C). This genomic deletion encompassed exon 12 of *OTOF*, which was the second genomic deletion detected ever from DFNB9 subjects to date. No capture failure or structural variation in and around the coding regions of *OTOF* was detected or suspected from SB42–79 in this second phase of TRS-134 analysis (data not shown), leaving molecular diagnosis of this subject still elusive.

## DISCUSSION

There are 2 isoforms of *OTOF*: long isoform, which uses exon 1 as a transcription start site, and short isoform, which uses exon 20. In addition, depending on the exon, which is used to encode the C-terminus and translation stop codon, 2 types of *OTOF* transcripts exist. In the human brain, 2 types of *OTOF* transcripts, which use either exon 47 or exon 48, have been detected.^18^ However, in human cochlea, only 1 type of *OTOF* transcript, which uses exon 48, exists exclusively; the *OTOF* transcript, which uses exon 47, does not exist.^26^ Consequently, the mutations in human cochlea, which reside in exon 48, cannot be compensated by *OTOF* transcript, which uses exon 47, and result in dysfunction of otoferlin. The p.R1939Q variant detected in this study resides in exon 48, and was reported in some studies to cause severe dysfunction of otoferlin and lead to ANSD.^24,27^ Therefore, it seems definite that p.R1939Q variant is a causative mutation of ANSD.

The prevalence of *OTOF* mutations in ANSD patients has been reported variously depending on the country: 86.7% (13 of 15) in Spain, 63.6% (7 of 11) in Brazil, 56.5% (13 of 23) in Japan, 55.6% (5 of 9) in USA, 22.7% (5 of 22) in Taiwan, and 6.8% (5 of 73) in China.^24,25,28–31^ A previous Korean study reported the prevalence of *OTOF* mutation in ANSD as 5.2% (1/19), which was much smaller than other countries.^32^ Those studies, except the Chinese study, included ANSD subjects without an imaging study, which can exclude anatomical abnormality of the cochlear nerve. In the Chinese study, only temporal bone CT, not internal auditory canal MRI, was used to evaluate the cochlear nerve. Therefore, considering the incomplete visualization of the cochlear nerve in temporal bone CT, there exists a possibility that several subjects with abnormality of the cochlear nerve were supposed to be included in such studies. These subjects could lead to an underestimation of the prevalence of *OTOF*.

Although both temporal bone CT and internal auditory canal MRI were performed in our previous study, it also showed that only one (SB4–11) of the 5 subjects (SH132–273, SB4–11, SB10–23, SB22–51, and SB42–79) exhibited any *OTOF* mutation.^33^ In the present study, we newly performed TRS-134 for 2 (SB22–51 and SB42–79) and reanalyzed the TRS data previously obtained from another 2 (SH132–273 and SB10–23) subjects. Finally, through this present study, we found *OTOF* mutations in 3 (SH132–273, SB10–23, and SB22–51) of the 4 subjects who had been considered not to be DFNB9 based on the previous study. We missed *OTOF* mutations in 3 DFNB9 subjects in our previous study.^33^ There are 3 possible reasons for this. The first reason may be that p.R1939Q was registered as SNP (rs201326023), not flagged SNP. We performed TRS or Sanger sequencing of *OTOF* to three subjects (SH132–273, SB10–23, and SB22–51) in the previous study. As p.R1939Q was registered as SNP, p.R1939Q detected in TRS and Sanger sequencing of *OTOF* was missed in the filtering process. In this study, we checked SNPs of *OTOF* gene obtained from TRS-134 one by one to prevent omitting candidate variants of *OTOF* gene. Then, we found the *OTOF* mutation in 2 subjects (SH132–273 and SB22–51). The second reason may be that structural variations, such as large deletions, cannot be detected solely by conventional Sanger sequencing. By incorporating additional phases of analysis on the TRS data which focused on the detection of structural variations, we were able to identify the possible large genomic deletion that encompassed exon 12, which was confirmed by breakpoint PCR (Fig. 4). When we detected 1 definitely pathogenic variant (p.P1939Q) from our ANSD patient (SB22–51), the presence of other occult variants within *OTOF*, such as a large genomic deletion or a variant residing in the regulatory sequences or intronic sequences of this gene in *trans* with the p.P1939Q allele, was strongly suspected. Considering the rarity of p.P1939Q among normal controls, the single heterozygous p.P1939Q allele detected in ANSD subjects is likely to indicate DFNB9 rather than a fortuitously detected variant. Our finding, a large genomic deletion in exon 12 of *OTOF*, was the second genomic deletion ever detected in DFNB9 subjects since the detection made by Zadro et al,^34^ who first reported the large genomic deletion in intron 18 of *OTOF* in 2010. In molecular genetic diagnosis using TRS, the heterozygote genomic deletion can be regarded as a low reading depth. Therefore, interpretation of low coverage (read depth) from TRS data always warrants caution. The third reason may be technical incompleteness of TRS. Next generation sequencing technology (NGS) allowed molecular genetic diagnosis to be more feasible and cost-effective, due to its high throughput characteristics. Especially, a big sized gene, such as *OTOF* comprising 48 exons, has been an obstacle for routine molecular diagnoses based on Sanger sequencing in a clinical setting, increasing the need for NGS. The coverage of TRS is usually expected to be deep enough to screen the target genes compared with whole exome sequencing, which should cover the whole human genes. However, a couple of recent studies still showed a risk of insufficient capturing of exons in TRS.^23,35^ Indeed, our previous study showed that 10% of target exons were not properly captured in TRS.^23^ Therefore, the coverage of TRS could not be enough in the experiments with many targets. In this study, exon 48 of *OTOF*, where the predominant variant p.R1939Q resides, was poorly covered in TRS, overall. This poor coverage for the first or the last exon of a certain gene is not uncommon.^36^ This could be due to the GC content. Sequentially, p.R1939Q was completely missed in the first phase of TRS data analyses of SB10–23 and SB204–398. Subsequent Sanger sequencing of exon 48 confirmed a homozygous p.R1939Q variant from SB10–23 and compound heterozygote p.R1939Q and p.R1856W from SB204–398. Taken together, our study showed caveats of solely relying on the primary filtering of TRS data as well as additional strength of employing TRS for etiologic diagnosis of ANSD in Koreans, warranting more cautious and multiphasic analyses of TRS data.

Taking our results into account, the prevalence of *OTOF* mutation in ANSD with the anatomically normal cochlear nerve was 85.7 % (6/7) in Korea. Contrary to previous reports in Koreans, we identify a strong etiologic homogeneity of the autosomal recessive or sporadic form of prelingual ANSD in case of the anatomically normal cochlear nerve in Koreans and now report *OTOF* mutations as the single overwhelming cause of it. Jeong and Kim reported that ANSD patients with normal radiological findings of the cochlear nerve in Korea showed excellent speech perception abilities after cochlear implantation.^37^ From these results, it can be assumed that the majority of ANSD with anatomically normal cochlear nerve may have functionally intact cochlear nerve, and solely be endocochlear hearing loss. This concept is consistent with our suggestion—strong etiologic homogeneity (*OTOF* mutation) of ANSD with anatomically normal cochlear nerve.

In addition, among the 14 alleles from 7 unrelated ANSD families in Korea, p.R1939Q was found in 50.0% of all alleles (7/14). The p.R1939Q was also found in 43.5% of all alleles (20 of 46) in Japanese ANSD patients.^24^ Furthermore, a different mutation in the same location (p.R1939W) was also reported in consanguineous Pakistani families.^26^ Therefore, p.R1939 seems to be a mutational hotspot. Consequently, *OTOF* mutation, especially p.R1939Q seems to be a predominant mutation in patients with prelingual ANSD with anatomically normal cochlear nerve. The p.R1939Q should be screened first in such patients in Korea to promote the cost-effectiveness of molecular genetic diagnosis (Fig. 1).

ANSD has heterogeneous etiologies. The outcomes of cochlear implantation for ANSD were reported to be diverse according to the etiology: natural recovery,^22,38–40^ poor results after cochlear implantation,^41–43^ and good results after cochlear implantation.^44–48^ Among several etiologies of ANSD, the *OTOF* mutation is a typical one, which is reported to show good results after cochlear implantation.^21^ Therefore, after the diagnosis of ANSD, it is crucial to identify *OTOF* mutations that support and justify bilateral early cochlear implantation. Based on our results, we propose a novel and comprehensive strategy incorporating imaging studies, screening of a prevalent mutation, and multiphasic analysis of TRS data in a stepwise manner (Fig. 1). This strategy would ensure more correct and effective molecular genetic diagnosis of DFNB9. Consequently, it would facilitate a timely auditory rehabilitation of DFNB9 subjects by enabling early bilateral cochlear implantation without unnecessary waiting. This would pave the way for the vitalization of “precision medicine” in the field of auditory rehabilitation for deaf subjects based on genetic etiology.^49^

In conclusion, we identify a strong etiologic homogeneity of the autosomal recessive or sporadic form of prelingual ANSD in case of the anatomically normal cochlear nerve in Koreans and now report DFNB9 as the single overwhelming cause of it. We also indicate that p.R1939Q is a predominant mutation and should be screened first in such patients in Korea to be cost effective in molecular genetic diagnosis. A more rigorous and multiphasic analysis of TRS data ensuring detection of capture failure and structural variations would be expected to reveal DFNB9 from a substantial portion of previously undiagnosed ANSD subjects in Korea. Usefulness of this comprehensive strategy may hold true for other deafness genes.
